# Magnetic reconnection in Saturn's magnetotail: A comprehensive magnetic field survey

**DOI:** 10.1002/2015JA022005

**Published:** 2016-04-08

**Authors:** A. W. Smith, C. M. Jackman, M. F. Thomsen

**Affiliations:** ^1^ Department of Physics and AstronomyUniversity of SouthamptonSouthamptonUK; ^2^ Planetary Science InstituteTucsonArizonaUSA

**Keywords:** magnetotail, Saturn, Cassini

## Abstract

Reconnection within planetary magnetotails is responsible for locally energizing particles and changing the magnetic topology. Its role in terms of global magnetospheric dynamics can involve changing the mass and flux content of the magnetosphere. We have identified reconnection related events in spacecraft magnetometer data recorded during Cassini's exploration of Saturn's magnetotail. The events are identified from deflections in the north‐south component of the magnetic field, significant above a background level. Data were selected to provide full tail coverage, encompassing the dawn and dusk flanks as well as the deepest midnight orbits. Overall 2094 reconnection related events were identified, with an average rate of 5.0 events per day. The majority of events occur in clusters (within 3 h of other events). We examine changes in this rate in terms of local time and latitude coverage, taking seasonal effects into account. The observed reconnection rate peaks postmidnight with more infrequent but steady loss seen on the dusk flank. We estimate the mass loss from the event catalog and find it to be insufficient to balance the input from the moon Enceladus. Several reasons for this discrepancy are discussed. The reconnection X line location appears to be highly variable, though a statistical separation between events tailward and planetward of the X line is observed at a radial distance of between 20 and 30*R*_*S*_ downtail. The small sample size at dawn prevents comprehensive statistical comparison with the dusk flank observations in terms of flux closure.

## Key Points

- New algorithm developed to find reconnection related events in spacecraft magnetometer data
- The 2094 events identified across the magnetotail at an average rate of 5.0 per day
- Reconnection X line location appears to be highly variable

## 1 Introduction

Magnetic reconnection is the fundamental process by which magnetic fields can reconfigure, and it plays a critical role in shaping planetary magnetospheres. Reconnection can release large amounts of the energy stored in the magnetic field, accelerating charged particles and dramatically changing the local magnetic topology. Reconnection also leads to the mixing of plasma from distinct magnetic regimes such as the solar wind and a planetary magnetosphere. In such a way reconnection has the potential for adding or removing mass from a planetary magnetosphere. In addition to changing the mass contained within the system reconnection also has the capacity to modify the amount of open flux contained within the polar caps. On the dayside of a planet it can open magnetic flux, connecting previously closed planetary field lines to the interplanetary magnetic field (IMF). Conversely, on the nightside of a planet reconnection can sometimes involve the closure of open field lines, those once connected into the IMF.

Reconnection and the resulting flows can be sampled in situ by spacecraft, by observing either the change in magnetic topology or the flows of plasma and charged particles. This study will concentrate on reconnection detected in situ within the magnetotail of Saturn. In the environment of a planetary magnetotail, reconnection has two main products that can be observed in situ: plasmoids and dipolarizations. On the planetward side of the X line previously stretched field lines recover to a more dipole‐like field arrangement following reconnection. This relaxation of the field is known as a dipolarization and has been observed by previous studies at Saturn [*Bunce et al.*, 2005; *Russell et al.*, 2008; *Thomsen et al.*, 2013, 2015; *Jackman et al.*, 2013, 2015]. Meanwhile, on the tailward side of the reconnection site the combination of plasma and field released downtail is called a plasmoid, first seen at Earth [*Hones*, 1976, 1977] and more recently detected at Saturn through data from the magnetometer and plasma instruments on the Cassini spacecraft [e.g., *Jackman et al.*, 2007; *Hill et al.*, 2008]. The effects of reconnection can be remotely observed as the changing magnetic field topology in the vicinity of the current sheet can cause the magnetotail lobe field lines to drape around passing structures. Such disturbances are known as traveling compression regions (TCRs) and can be observed both planetward and tailward of the reconnection location [*Slavin et al.*, 1984, 1993, 2005].

Tail reconnection was first observed in situ at Saturn during the outbound pass of Cassini's Saturn Orbit Insertion maneuver in 2004. *Bunce et al.* [2005] observed an event in magnetometer, plasma, and radio wave data consistent with a dipolarization of the field following solar wind compression‐induced tail reconnection. The deepest tail orbits performed in 2006, where Cassini traveled up to 68*R*_*S*_ downtail (1*R*_*S*_=60268km), have provided the most heavily studied data in terms of reconnection studies. *Jackman et al.* [2007, 2008] and *Hill et al.* [2008] first reported several observations of encounters with plasmoids and dipolarizations during these 2006 orbits, identifying them from both magnetic field and plasma data. The catalog of reconnection events was later expanded by *Jackman et al.* [2011] to include 34 newly discovered plasmoid encounters. This work also began to consider the impact of these events on the open flux contained in the polar cap, by examining the statistical post plasmoid plasma sheet (PPPS), a region where flux is closed following plasmoid release [*Richardson et al.*, 1987]. Most recently, the 2006 data were reevaluated in detail by *Jackman et al.* [2014] who reported a total of 69 plasmoids, 17 TCRs, and 13 planetward moving structures. From this enlarged sample of events it was possible to estimate the mass loss from the Kronian magnetosphere due to plasmoid loss down the magnetotail. It was found, as in previous work, that plasmoid loss through large‐scale events was insufficient to account for the mass entering into the system from Enceladus and other sources [*Bagenal and Delamere*, 2011]. Solutions put forward to solve this problem include unobserved loss down the flanks of the magnetosphere, discussed for the Jovian magnetosphere by *Kivelson and Southwood* [2005], or the loss of mass through small‐scale mechanisms, such as cross‐field diffusion [*Bagenal and Delamere*, 2011]. Most recently, *Cowley et al.* [2015] developed a theoretical argument suggesting that the event duration, as defined in recent studies, only represents a small portion of the plasmoid structure. In previous work the duration of the event is generally defined as the time between the peak northward and peak southward field, following the method of *Slavin et al.* [1993]. *Cowley et al.* [2015] argued that this measure only calculates the length of time that the spacecraft spends within some part of the structure (depending on the relative trajectory) and that this may only be a small planetward fraction of the full extended plasmoid. This could reduce (or explain) the mass imbalance and decrease (or remove) the requirement for small scale or hidden mass loss mechanisms.

Over the years various different theoretical models have been proposed for the convection patterns within the Kronian magnetosphere [*Cowley et al.*, 2004; *Kivelson and Southwood*, 2005; *Kane et al.*, 2014; *Delamere et al.*, 2015]. Though different in some respects, all models suggest differences between the dawn and dusk flanks of the magnetotail, differences that have been observed in both plasma and magnetic field data [*Arridge et al.*, 2015a, and references therein]. For example, evidence suggests that the flows are predominantly corotational throughout most of the magnetotail inside ∼50*R*_*S*_, with outflow common from dusk to ∼0200–0300 local time and some inflow seen at local times beyond 0200 [*McAndrews et al.*, 2009, 2014; *Kane et al.*, 2014; *Thomsen et al.*, 2013, 2014]. Asymmetries are also present in some global MHD [*Jia et al.*, 2012] and multifluid models [*Kidder et al.*, 2012]. As mentioned above, all previous studies of reconnection from a magnetometer perspective have focused on Cassini's deepest tail orbits during 2006, which spanned local times from 22:00 to 06:00 h [e.g., *Jackman et al.*, 2014]. Thus, they are somewhat limited in terms of their ability to examine dawn‐dusk asymmetries.

The aim of this study is to expand upon previous reconnection surveys, using a new automated technique to identify reconnection events from Cassini magnetometer data and incorporating new data from the dusk orbits of 2009 and 2010 in addition to the midnight‐dawn orbits of 2006 to gain fuller local time coverage of the magnetotail. This selection of data also allows the examination of reconnection both preequinox and postequinox. The new technique enables more consistent and unbiased event identification and includes smaller scale events missed by previous studies. The inclusion of data surveying different regions of the magnetosphere enables more reliable conclusions to be drawn regarding the location and occurrence of reconnection in the Kronian magnetotail.

Section 2 below introduces the data set, field deflections, and definitions. This is followed in the next section by a detailed discussion of the algorithm developed to find the reconnection related events. Section 4 then explores the catalog of detections and some of the broad statistical properties. The occurrence frequency, location, and signatures are then discussed in section 5 before section 6 looks into the more global interpretation of the results.

## 2 Data Set and Observations

The data primarily used in this study come from the Cassini magnetometer [*Dougherty et al.*, 2004], in orbit around Saturn since July 2004. The coordinate system selected for this study is the Kronocentric radial theta phi (KRTP) system. In this spherical polar system the radial component (*B*_*r*_) is positive outward from Saturn, the meridional component (*B*_*θ*_) is positive southward (at the equator), and the azimuthal component (*B*_*ϕ*_) is positive in the direction of corotation (prograde). This coordinate system was evaluated by *Jackman et al.* [2009] and shown to be useful in distinguishing reconnection related events from those caused by waves in the hinged current sheet. One minute resolution data were selected as appropriate for this work; previous studies have shown the average duration of a reconnection related event in the Kronian magnetotail to be around 18 min [*Jackman et al.*, 2014].

### 2.1 Data Location

**Figure 1:**
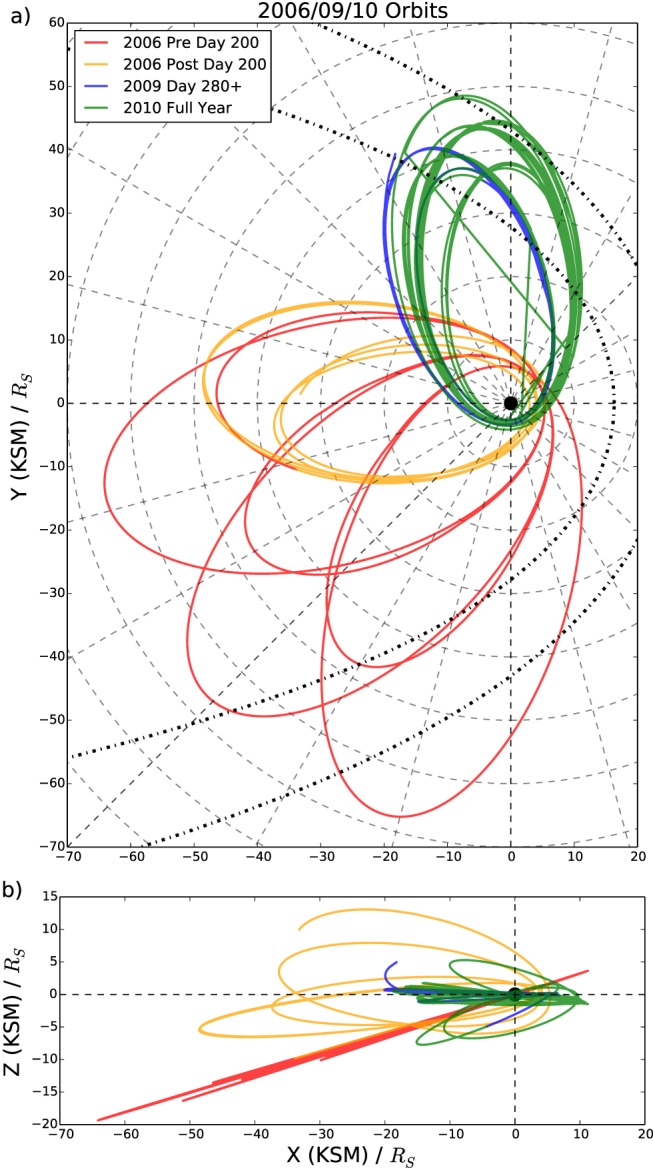
Cassini trajectory during 2006, 2009, and 2010 in the KSM (Kronocentric Solar Magnetospheric) coordinate system; the *x* axis traces the sunward direction, the *x*‐*z* plane contains the planetary dipole axis, and the *y* axis is positive toward dusk. (a) The *X*‐*Y* view. (b) The *X*‐*Z* projection. The dashed concentric circles on Figure 1a are marked every 10*R*_*S*_, while the radial dashed lines identify the local time hours. The orbital trajectory is marked in red for orbits before day 200 in 2006, orange for orbits after day 200 in 2006, blue for orbits after day 280 in 2009, and green for orbits during 2010. The black dash‐dotted lines marked Figure 1a show the *Kanani et al.* [2010] model magnetopause for solar wind dynamic pressures of 0.1 and 0.01 nPa.

Figure 1 shows the Cassini orbits examined in this study. They are displayed in the KSM (Kronocentric Solar Magnetospheric) coordinate system where the *x* axis points toward the Sun, the *x*‐*z* plane contains the planetary dipole axis, and the *y* component completes the right‐handed set. Reconnection is thought to occur at the center of the hinged current sheet, a key consideration when selecting appropriate data. Data from 2006 have been heavily relied on in the past for reconnection studies; during 2006 Cassini performed its deepest tail orbits around midnight and dawn. Cassini began 2006 with equatorial orbits around the dawn flank (shown by the red orbits in Figure 1), moving to slightly inclined orbits around midnight later in the year beyond day 200 (the orange orbits in Figure 1). To complement this data, we use data from orbits in late 2009 (day 280 onward, shown by the blue orbits in Figure 1) and 2010, giving equatorial coverage on the dusk flank (the green orbits in Figure 1). During early 2009 Cassini performed high‐latitude passes of the dusk flank, and it was not until later in that year that the orbit became more equatorial and suitable for this study. These low‐latitude relevant dusk orbits continued throughout 2010. The 2009–2010 orbits sample the majority of the dusk flank, but the orientation of the orbits only allows exploration of distances up to 20*R*_*S*_ down the magnetotail. These newly included orbits sample local time magnetotail regions between 18:00 and 22:00, filling the region of the magnetotail unexplored during 2006.

### 2.2 Event Field Deflections

**Figure 2:**
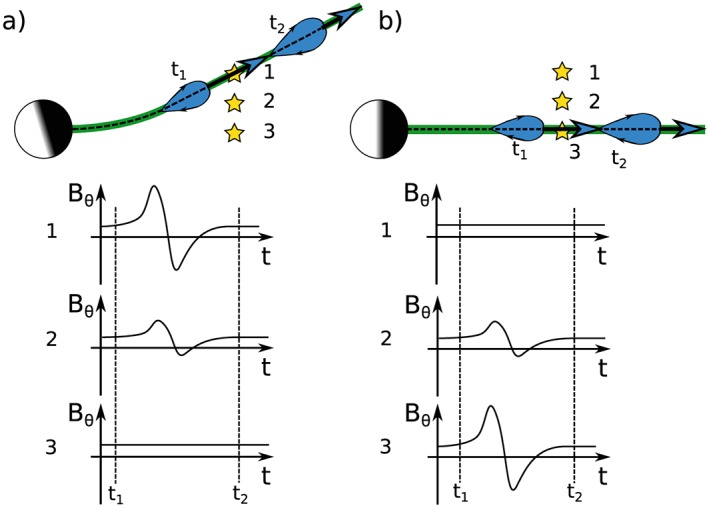
Schematic showing model magnetometer deflections associated with plasmoid release downtail. The magnetometer sketches are shown for spacecraft at three different latitudes; the spacecraft positions are represented by stars. The current sheet is indicated with a black dashed line, while the green region indicates the extent of the plasma sheet. (a) The case when the current sheet is hinged up out of the equatorial plane. (b) The plasma sheet is in the equatorial plane. The (blue) plasmoid is shown propagating down the current sheet at two times, *t*_1_ and *t*_2_, with the direction of travel given by the large blue arrows. The small black arrows on the plasmoids indicate the magnetic field direction around the structure.

*Jackman and Arridge* [2011] used magnetic field data in the KRTP system to show that the average north‐south component of field in the Kronian magnetotail was small and southward during the deep, relatively low latitude orbits of 2006. Any deviation from this southward steady state may be caused by reconnection at some location in the magnetotail. At Saturn, tailward moving events are expected to display a characteristic south to north deflection of the field. Figure 2 shows examples of the possible field deflections caused by such structures. It is important to note that although the south to north deflection of the field has been related to tailward (radially outward) moving events there could be a considerable azimuthal/corotational component to their motion following release. This is seen consistently in both statistical plasma flow maps and magnetic field studies [*McAndrews et al.*, 2009, 2014; *Kane et al.*, 2014; *Thomsen et al.*, 2014; *Jackman et al.*, 2014], where the vast majority of plasma flow is in the direction of corotation. However, for the purpose of this paper the term tailward (planetward) moving should be understood as events inferred to be tailward (planetward) of the reconnection site, from the orientation of the *B*_*θ*_ deflection.

The field structure of plasmoids can, broadly speaking, take one of two morphologies: closed loops or helical fields (flux ropes) [*Slavin et al.*, 2003; *Eastwood and Kiehas*, 2015, and references therein]. These categories can be principally identified from inspection of the *B*_*ϕ*_ and |*B*| components of the field; loop‐like plasmoids show a strong reduction of the field at the center of the structure while flux rope type plasmoids show a strong intensification of both the *B*_*ϕ*_ and |*B*| components of the field at the core of the helical fields. These field changes are distinct from TCRs, which display a smooth increase in the total field corresponding to the compression of the field due to the plasmoid's passage downtail. Planetward moving events meanwhile are expected to show the opposite bipolar field signature, a north to south deflection, associated with the snapping back of recently broken field lines to a more dipole‐like configuration. Though dipolarizations exhibit a dip in the north‐south field component at the leading edge of the event the field does not always fully reverse (turn northward, or negative in the KRTP system, at Saturn) [*Ohtani et al.*, 2004; *Shiokawa et al.*, 2005; *Nakamura et al.*, 2009; *Pan et al.*, 2015].

It should be noted that there are other phenomena that can cause variation in the north‐south component of the field. These include waves or flapping of the plasma sheet and passage of the spacecraft through Saturn's low‐latitude boundary layer (LLBL) or magnetopause. Further discussion of these can be found in section 3.1.

### 2.3 Viewing Region Definition

When looking at the occurrence rate or frequency of reconnection‐related events, the spacecraft “viewing region” must be defined. This is the area of the magnetosphere within which events can be detected in situ by the spacecraft. In this study it has been defined as anywhere on the nightside of Saturn, at a distance of more than 15*R*_*S*_ from the planet and within the magnetosphere. We also require that Cassini is not performing SCAS (Science Calibration and Alignment Subsystem) maneuvers, and the field measured by Cassini is unaffected by Titan. No latitude criteria have been enforced, first because of the different seasons during which the data were collected and second because this has been accounted for in the selection of the orbits used by this study. Times outside the magnetosphere (and data within 15 min of the crossing) were excluded using a database of magnetopause crossings, the same utilized by *Pilkington et al.*[2015].

### 2.4 Magnetospheric Environment Classification

The location of the spacecraft relative to the plasma sheet has implications for the interpretation of the occurrence rate of events, i.e., a low frequency measured in the lobe may only relate to those events large enough to trigger detections far from the current sheet center. For this purpose, and also for automatically classifying events as plasmoids or TCRs, criteria based on the magnetic field readings were developed to constrain Cassini's local plasma environment. Previous studies that have attempted similar classifications have additionally used criteria on the readings from the Cassini Electron spectrometer (ELS) instrument, a constituent of the Cassini Plasma Spectrometer (CAPS) [*Young et al.*, 2004]. ELS measures electrons over the energy range 0.8eV to 27keV and can be useful when differentiating the high‐density current sheet from the very low plasma densities common in the magnetotail lobes [*Arridge et al.*, 2011; *Jackman and Arridge*, 2011]. However, due to pointing constraints and low counts in the lobes, no formal criteria have been applied to the ELS readings, though example periods were compared to the magnetic field conditions to determine the correct criteria. The field criteria are similar to those used in previous studies, for example, those utilized by *Simon et al.*[2010] and *Jackman and Arridge* [2011]. Equations (1) and (2) show the two criteria that must be satisfied for Cassini to be classified as within the lobe. These criteria are checked during events, and if they are satisfied for the entire event duration, then the event is classed as a TCR. If either condition is violated during the event, then it is deemed to be close to the plasma sheet.

(1) $${}^{\left|B_{r}\right|}{/}_{\left|B\right|}\geq 0.6$$

(2) $${}^{\sigma B_{r}}{/}_{\left|B\right|}\leq 0.25$$

where *σ* *B*_*r*_ is the standard deviation of the *B*_*r*_ component of the field for the period 30 min either side of the data point. The conditions were selected as, from a magnetic field perspective, the lobe can be characterized by quiet steady field predominantly in the radial direction. During the process of selecting the limits outlined above it was noted that some borderline events see quiet, radial field, but small counts in the ELS data. The resulting TCR classifications were deemed acceptable, as no significant changes in plasma character were observed during the events. The lack of a sharp boundary between lobe‐like and current sheet‐like field character was also noted by *Jackman and Arridge*[2011].

Equations (1) and (2) have also been applied to the data in general to classify Cassini's local environment during all orbits. As with the event classification, if both criteria are satisfied, then Cassini is said to be within the lobe; if either is violated, then Cassini is classified as near to the plasma sheet.

### 2.5 Magnetospheric Environment Variation Within Data Set

It is important to consider the effect of changing Kronian season on the magnetospheric environment encountered by Cassini during the 2006, 2009, and 2010 orbits. 2006 corresponded to Southern Hemisphere summer, and as a result the hinged current sheet was displaced above the dipole equator, generally above a latitude of ∼5°. The warping and hinging of the Kronian current sheet is discussed in detail by *Arridge et al.* [2008]. The arrangement during Southern Hemisphere summer is shown schematically in Figure 2a, where yellow stars represent possible positions of the spacecraft (at different latitudes). Accordingly, the early 2006 low latitude (∼0°) dawn orbits found Cassini mainly within the southern lobe of the magnetotail (similar to position 3 in Figure 2a); Table 1 shows that 92% of the early part of the year was spent within the lobe. Later in 2006, around day 200, the orbit became more inclined and more similar to positions 1 and 2 in Figure 2a. Consequently, Cassini spent more time within the plasma sheet; from Table 1 the lobe occupation time dropped to 74%. In comparison the 2009 and 2010 data were recorded around and beyond Kronian equinox, a time when the plasma sheet was located in a more equatorial location, similar to the position shown in Figure 2b. Therefore, the low‐latitude orbits of late 2009 and 2010 lay within the plasma sheet the majority of the time (similar to position 3 in Figure 2b), with Cassini spending 56% and 59% of the time near the plasma sheet respectively (from Table 1). As plasmoids are thought to form and propagate along the current sheet, orbits located within the lobe are likely to see only the largest events, either as they cause the plasma sheet to bulge over the spacecraft allowing direct plasmoid detection or as the downtail motion of plasmoids warps the surrounding lobe field lines such that TCRs can be detected from locations in the lobe.

**Table 1:** Data Summary

	Viewing Region (Days)	Lobe (Days)^a^	Plasma Sheet (Days)^a^
All Years	415	265 (64%)	150 (36%)
2006	208	177 (85%)	31 (15%)
2006 Preday 200	131	121 (92%)	10 (8%)
2006 Postday 200	76	56 (74%)	20 (26%)
2009	50	22 (44%)	28 (56%)
2010	157	65 (41%)	92 (59%)

a Location inferred from criteria on the magnetometer readings outlined in section 2.4.

## 3 Algorithm for Event Identification

An algorithm was designed to automate the search for reconnection related events (plasmoids, dipolarizations, and TCRs), creating an unbiased and consistent catalog. The main criterion is a clear, unambiguous deflection of the north‐south component of the field, unrelated to the periodic or long‐term variation observed during orbits.

At this point it is important to mention that though Figure 2 shows symmetric bipolar deflections of the field, they are not always observed. The classic, symmetric, bipolar signature is only seen if the spacecraft trajectory passes through both the leading and trailing portions of the plasmoid evenly. In comparison, if the spacecraft trajectory is such that the passage through a plasmoid structure misses the leading edge or occurs at a large distance from the center, then the signature is more unipolar [*Borg et al.*, 2012; *Jackman et al.*, 2014]. Such magnetic field signatures were discussed in detail by *Cowley et al.* [2015], who suggested that the coverage of Cassini often precludes the observation of the full length of plasmoid structures.

### 3.1 Event Selection Process

**Figure 3:**
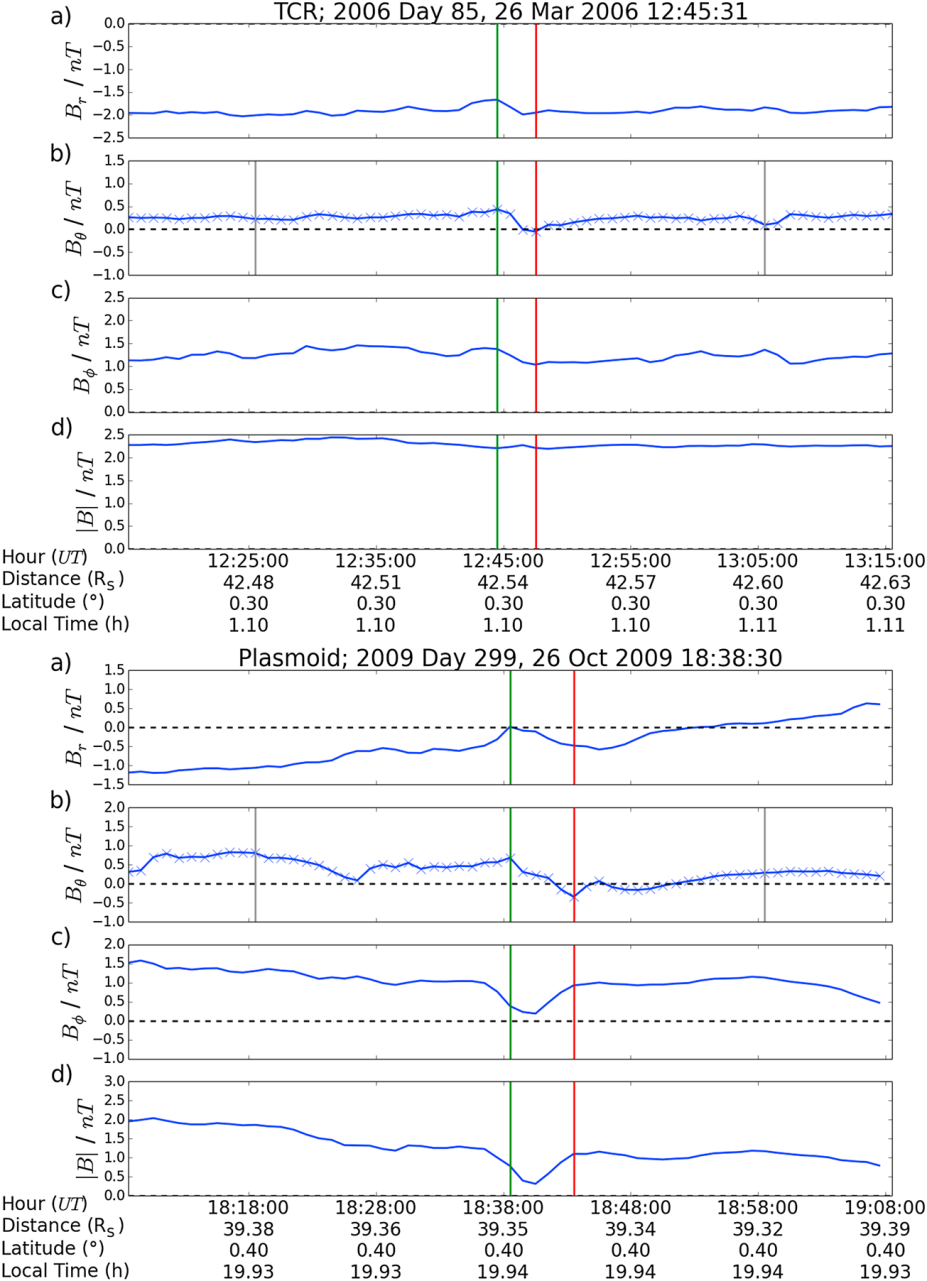
Algorithm outputs showing example (top) TCR and (bottom) plasmoid detections. The magnetic field is given in KRTP coordinates. The green and red lines give the algorithm's defined start and end of the events, respectively. The faint grey vertical lines give the extent of the time windows (20 min) either side of the event that are searched for the beginning and end of the event.

Two example outputs from the algorithm are provided in Figure 3. The top example shows a TCR observed in 2006, while the second example shows a plasmoid observed during 2009. Neither example was selected by previous reconnection surveys.

The algorithm first takes the average of *B*_*θ*_ for the 30 min either side of each data point. This average is taken to provide a baseline about which a deflection in *B*_*θ*_ must be observed. Every occasion where the data cross through the baseline is then recorded (henceforth referred to as a *B*_*θ*_ crossing). These provide locations at which the algorithm can check for significant deflections.

The algorithm searches for the start and end of the deflection within a 20 min time window either side of each *B*_*θ*_ crossing. The aim of this step is to select the local maxima and minima of *B*_*θ*_ as the start and end of the event, as in *Slavin et al.* [1993] and more recently *Jackman et al.* [2014] and *Vogt et al.* [2014]. The 20 min window was created to balance minimizing the computational time while allowing the correct limits to be found for known events [from *Jackman et al.*, 2014].

If the *B*_*θ*_ deflection is northward, i.e., the value of *B*_*θ*_ decreases, then local maxima of *B*_*θ*_ (places where the *B*_*θ*_ value is greater than at adjacent time points) up to 20 min before the *B*_*θ*_ crossing are selected as potential starts. On the other side of the *B*_*θ*_ crossing the local minima of *B*_*θ*_ (where the *B*_*θ*_ values are less than at their adjacent time points) are selected as potential ends. The opposite holds true if the deflection is southward, i.e., has increased the value of *B*_*θ*_.

All pairings of potential starts and stops are then checked. A second‐order polynomial is least squares fitted between each pairing. The value of *r* ^2^ (the coefficient of determination) is calculated for each fit using equation (3).

(3) $$r^{2}=1{-}^{\overset{N}{\underset{0}{\sum }}{\left(B_{\theta }^{t}-f_{t}\right)}^{2}}{/}_{\overset{N}{\underset{0}{\sum }}{\left(B_{\theta }^{t}-\bar{B_{\theta }}\right)}^{2}}$$

where *t* = 0 is the start and *t* = *N* is the end, $B_{\theta }^{t}$ is the value of *B*_*θ*_ for point *t*, $\bar{B_{\theta }}$ is the average of *B*_*θ*_ between the start and end, and *f*_*t*_ is the value of the fitted polynomial for point *t*. If the value of *r* ^2^ calculated is less than 0.9 (the quality of the fit to the data is poor), then the start/end pair is rejected. For all pairs that have a value of *r* ^2^≥0.9 a value of Δ*B*_*θ*_ is calculated (the “size” of the event), using equation (4).

(4) $$\Delta B_{\theta }=B_{N}-B_{0}$$

The start/end pair with the highest absolute value of Δ*B*_*θ*_ is then selected. The first condition on Δ*B*_*θ*_ for the event to be accepted is

(5) $$\frac{\left|\Delta B_{\theta }\right|}{{B_{\theta }}_{\text{RMS}}}\geq 1.5$$

where the RMS (root‐mean‐square) of *B*_*θ*_ is calculated for a period extending 30 min either side of the *B*_*θ*_ crossing. This criterion was selected, as it was shown to preferentially select only significant deflections. The second criterion on event acceptance is that |Δ*B*_*θ*_|≥0.25nT. This lower limit was imposed after inspection of a selection of false positive detections. The time window for which the RMS is calculated is the full time plotted on Figure 3. The vertical grey bars show the maximum possible length of the event.

This method produces a list that often contains duplicates and pairs of events. Duplicates are cases where the events have the same orientation and overlap. “Pairs” are, for example, a northward turning immediately followed by a southward turning. This combination can occasionally be erroneously interpreted by the algorithm as a tailward moving plasmoid followed immediately by a dipolarization; however, the correct interpretation (in this example) is that of a tailward moving plasmoid followed by the return of the field to background (small and southward) levels. To deal with these, a series of rules were developed. Any events that overlap or occur with the opposite orientation within one duration (difference in time between the start and the end of the event) of the start or the end of the event are grouped together. The event with the largest Δ*B*_*θ*_ in the group is selected. If another in the group has a value of Δ*B*_*θ*_ within 10% of the largest event, then they are inspected by eye to pick the correct deflection. In the vast majority (>95%) of cases the largest detection was confirmed by eye to correspond to the correct deflection.

The events that pass all of the above criteria were then inspected by eye, and the false detections were removed. False detections are due to the phenomena mentioned in section 2.2: mainly flapping of the plasma sheet and encounters with the magnetopause or LLBL. Observing the field over a period of several days around the identified event allowed those related to the bulk flapping motion of the current sheet to be distinguished; they are related to longer‐term quasi‐sinusoidal trends in the north‐south component of the field [*Nakagawa and Nishida*, 1989; *Jackman et al.*, 2009]. Magnetopause crossings, on the other hand, were discarded using the method discussed in section 2.3. Encounters with the LLBL can be typified by a clear change in the field regime either side of a large, rapid rotation of the field [*Masters et al.*, 2011].

During the inspections several events were found to have been detected with the opposite orientation to that which may have been chosen by eye. They were noted (and appear in Table 2) but do not appear in the rest of the analysis.

**Table 2:** Algorithm Results

	Tailward Events	Planetward Events	Incorrect Orientation	False Positives	Clusters (Isolated)
Total Detections	1382 (58%)	712 (30%)	72 (3%)	222 (9%)	381 (220)
2006	693 (59%)	354 (30%)	21 (2%)	105 (9%)	171 (74)
2006 (Preday 200)	187 (64%)	85 (29%)	6 (2%)	13 (5%)	65 (47)
2006 (Postday 200)	506 (57%)	269 (31%)	15 (2%)	92 (10%)	106 (27)
2009 (After day 280)	158 (55%)	87 (30%)	8 (3%)	35 (12%)	55 (42)
2010	531 (57%)	271 (29%)	43 (5%)	82 (9%)	155 (104)

### 3.2 Failed Criteria

Many different criteria were tested and later discarded in favor of the conditions outlined above. For example, a threshold on Δ*B*_*θ*_ was tested that varied with radial distance, a similar function to the way the lobe field reduces with radial distance down the tail [*Jackman and Arridge*, 2011]. This was found to give some good results; however, as it was not dependent on the local properties of the field it missed many of the small‐scale events, especially TCRs, that this study targeted.

Another threshold that was tested involved a limit that was linked to the mean absolute value of *B*_*θ*_. This gave reasonable results, but it showed no distinct advantages over the threshold given in equation (5).

Another technique considered in place of the algorithm was a wavelet analysis/fitting method. This requires a base “wavelet” which is manipulated to attempt to match the data, and the quality of the fit is then evaluated. This method was discarded, as it may have too tightly constrained the type of event that was found. For example, there are two similar, but distinct, signatures/types of *B*_*θ*_ deflection observed depending on whether open flux is closed in the course of the reconnection event, discussed in detail by *Jackman et al.* [2011]. Additionally, the method involves fitting the template wavelet for a range of timescales; occasionally, the best fit found was not the same as the event that would be identified by eye. This result could be due to degeneracies in the wavelet fit, where one or more length scales satisfactorily fit the data and the technique simply picks the incorrect one.

Overall, the algorithm presented in section 3.1 represents an efficient method to automate the search for reconnection events in a planetary magnetotail.

## 4 Catalog

In total, the algorithm made 2388 detections in the 2006, 2009 (day 280 onward), and 2010 Cassini magnetometer data. A summary of the algorithm detections is presented in Table 2. Of the 2388 detections, 2094 (88 %) were confirmed by eye as likely corresponding to reconnection events. This leaves 294 events (12 %) which were rejected. Of these rejections, 72 (24% of the subset) are believed to be detections of reconnection related events, but with the incorrect orientation (and so represent the return of the field to normal levels or the run‐up to an event), where the “correct” detection was erroneously excluded by the process outlined in section 3.1. With this in mind the false positive fraction could be said to be around 9% (222 Events). The false positive events were thought to be caused by encounters with the LLBL or flapping magnetotail current sheet. All figures and analysis from this point will focus solely on those events confirmed by eye. This large number of events provides an excellent base for statistical analysis. Importantly, the data set covers a large range of local times, radial distances, latitudes, and Kronian season, allowing the differences to be explored.

Of the 86 events published in the previous study by *Jackman et al.* [2014], 77 (90%) were independently refound by the algorithm. Further inspection of the events not found revealed that several are rejected by the exclusion of data around magnetopause crossings (±15 min of *Pilkington et al.* [2015] magnetopause encounters).

Recently, *Arridge et al.* [2015b] uncovered an encounter of the Cassini spacecraft with a reconnection diffusion region. During this encounter they identified several secondary islands, in addition to the crossing of the X line itself. The algorithm recovers three tailward detections before the crossing of the X line, in agreement with their identification of secondary islands. It then recovers the X line crossing itself, along with a further secondary island some time after. A detailed search of the catalog for further diffusion region encounters will form the basis of future work.

The final column of Table 2 shows the number of event “clusters” identified. A cluster has been defined as a group (or chain) of events that occur within 180 min of their nearest neighbor, as in *Jackman et al.*[2014] (scaled for the Saturn system from the value of 30 min used by *Slavin et al.* [1993]). It can be seen that although 2094 detections were made, in total, there were 381 groups of more than one event. These clusters generally consisted of 2–6 events. Only 220 of the events were found to occur in isolation. For example, all five detections relating to the diffusion region identified by *Arridge et al.* [2015b] are classified as one cluster. In the same manner, the chain of events highlighted by *Jackman et al.* [2014, Figure 5], where they see a procession of four tailward events, is classified as one cluster. So the results of the algorithm can be thought of as relating to at least 601 “episodes” of reconnection (381 clusters + 220 isolated events).

Table 3 displays some of the statistical properties of the event catalog. Of the 2094 events identified by the algorithm 66% are inferred to be tailward of the X line, from the orientation of the field deflection. In theory, given appropriate viewing geometry, symmetric either side of a reconnection X line, equal numbers of each orientation of event should be observed, assuming reconnection events produce both a plasmoid (tailward) and dipolarization (planetward). The imbalance between the detections could suggest that Cassini is more often tailward of the reconnection site during the orbits selected.

**Table 3:** Event Details

	All Events	Tailward Events		Planetward Events	
Number of Events	2094	1382 (66%)		712 (34%)	
Average Duration (min)	7.8	9.2		5.3	
Average |Δ*B*_*θ*_| (nT)	0.98	0.96		1.01	
		Near Plasma		Near Plasma	
		Sheet	TCR	Sheet	TCR^a^
Number of Events	2094	1011	371	484	228
Mean Duration (min)	7.8	8.6	10.7	5.6	4.6
Median Duration (min)	6.0	6.0	8.0	4.0	4.0
Durations Range (min)	1–36	1–35	1–36	1–31	1–20
Mean |Δ*B*_*θ*_| (nT)	0.98	1.07	0.67	1.16	0.69
Median |Δ*B*_*θ*_| (nT)	0.83	0.94	0.57	0.99	0.59
|Δ*B*_*θ*_| Range (nT)	0.25–4.71	0.25–4.24	0.25–2.22	0.26–4.71	0.25–2.81
Radial Distance Range (*R*_*S*_)	15.0–68.3	15.5–68.2	18.5–68.3	15.0–65.0	16.6–68.2
Local Time Range (h)	18.05–5.12	18.02–5.11	18.20–5.11	18.02–4.12	18.29–4.49

a Distinctions made using the criteria on the magnetometer readings outlined in section 2.4.

## 5 Occurrence, Location, and Morphology

### 5.1 Event Occurrence and Location

From Table 3 it can be seen that events are, in general, observed at all local times and radial distances sampled by Cassini. However, we now examine any statistical dependence of events on location and season.

#### 5.1.1 Event Occurrence

**Figure 4:**
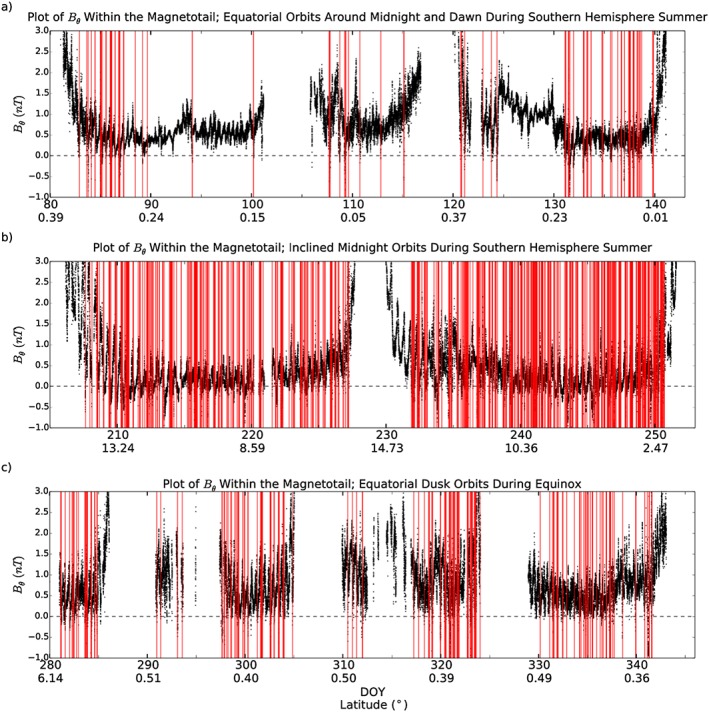
Plot showing the variation in the north‐south component of the magnetic field (*B*_*θ*_ in KRTP coordinates) during selected orbits. Each panel of the plot shows several orbital rotations of Cassini between perikrones. Times when Cassini was outside of the magnetosphere, near Titan or performing a SCAS interval, have been removed from the data. Red vertical bars represent reconnection events identified by the algorithm and then confirmed by eye. (a) Equatorial orbits during Southern Hemisphere summer around dawn and midnight during which time Cassini was mostly within the lobe. (b) More inclined orbits during Southern Hemisphere summer around the midnight local time sector. (c) Equatorial dawn orbits during equinox.

Figure 4 shows plots of the north‐south component of the field at 1 min time resolution over several orbits, with the confirmed event detections marked on as vertical bars. Each panel has been selected to show different seasons, latitudes, or local times. Figure 4a shows the early 2006 low‐latitude dawn flank orbits, recorded during Southern Hemisphere summer (where Cassini was in similar locations to positions 2 and 3 of Figure 2a). Figure 4b shows the orbits from later in 2006 during which time the midnight region of the magnetosphere was explored at a slightly higher latitude (similar to positions 1 and 2 in Figure 2a). Finally, Figure 4c shows dusk flank equatorial orbits performed in 2009, around Kronian equinox (where Cassini was found in a similar environment to position 3 in Figure 2b). Any data recorded when Cassini was outside of the magnetosphere, close to Titan, within 15*R*_*S*_ of the planet or during a SCAS interval, have been removed. Examples of intervals where data were removed on Figure 4a include between days 102 and 106 (where Cassini was within the magnetosheath) and between 117 and 119 (where Cassini was near perikrone and closer than 15*R*_*S*_). Each panel of the plot shows several orbits of Cassini between the points of closest approach to Saturn.

Plotting the catalog of events in this manner helps to highlight a few key aspects. First, a regular oscillation of *B*_*θ*_ can be seen in all panels. This is likely related to the flapping of the hinged current sheet around and perhaps over the spacecraft, discussed in detail by *Arridge et al.* [2008, 2011] and *Jackman et al.* [2009]. Second, there is a clear change in both the number and frequency of events between the different magnetospheric environments explored by Cassini. Figure 4a, detailing dawn flank equatorial orbits performed during Southern Hemisphere summer, shows a low occurrence rate and an uneven distribution of event detections. During these orbits only around 8% of the time was spent within the plasma sheet (from Table 1). The low occurrence rate could be evidence of sporadic reconnection between midnight and dawn. The patchy nature of the observations could also be a result of the variable distance to the plasma sheet over this period, as the plasma sheet flaps up and down near the spacecraft. When Cassini is closer to the plasma sheet, it could become more likely to observe reconnection products.

An interesting period included in this plot is around day 127 (Figure 4a, right). For a few days around day 127 the field strength increases, with little reconnection observed. This buildup is then lost, accompanied by a flurry of reconnection events. *Jackman et al.* [2010] related this field signature to the buildup of open flux in the tail lobes following solar wind compression induced dayside reconnection. This open flux is later closed through a cascade of reconnection events; many of which are picked up by the algorithm. Recently, a solar wind compression event has been shown to result in sustained lobe reconnection, inferred from the plasma sheet composition [*Thomsen et al.*, 2015].

Comparatively, Figure 4b shows a much larger number and frequency of events. The orbits shown here are more inclined (with latitudes up to 15°) and center around midnight. Due to the hinging of the current sheet at Saturn during Southern Hemisphere summer Cassini is more often located within the plasma sheet during these orbits (lobe residence fraction decreases from 92% to 74% compared to the orbits displayed in Figure 4a). Figure 4b contrasts once again with Figure 4c, which shows equatorial dusk flank observations (during equinox), again mostly within the plasma sheet, but with a lower rate of observation than Figure 4b. The events in Figure 4c are also seen more consistently and appear less clustered than they are in Figure 4a.

The difference between Figures 4a and 4b could be explained by the different magnetospheric environments encountered, i.e., the latitude differences illustrated by positions 1, 2, and 3 in Figure 2a. If this is the case, then it suggests that the in situ viewing conditions are highly latitude dependent, and thanks to the hinging of the current sheet, season dependent. Comparing Figures 4b and 4c, the main difference in orbit is the local time region explored, midnight in Figure 4b and dusk in Figure 4c). The latitude difference is compensated for with the seasonal change, meaning that both data sets are collected close to or within the plasma sheet. This could suggest an increased frequency of events around the midnight region of the magnetotail, with more infrequent but steady loss down the dusk flank. The dependence on distance to the plasma sheet will be explored in the next section.

**Figure 5:**
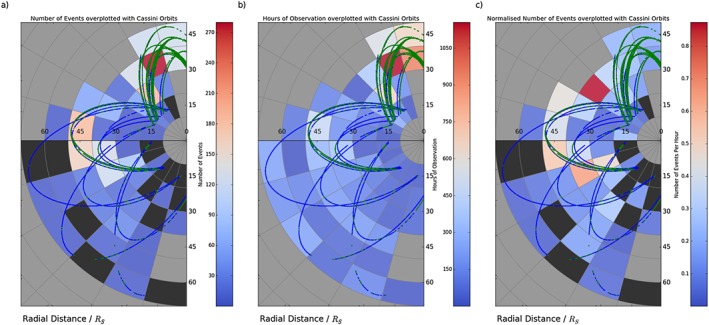
Color coded maps with 10*R*_*S*_ radial and 1 h local time bins. Maps are shown projected on the equatorial plane of the KSM system, with the Sun off to the right. Superimposed on the figure are the orbital trajectories of Cassini for 2006, late 2009, and 2010, within the viewing region. The blue sections of the trajectory represent times identified as within the lobe, while the green orbits outline times when Cassini is closer to or within the plasma sheet (from the criteria in section 2.4). Black regions show sectors where data were collected within the viewing region, but no events were detected. Meanwhile, grey segments show areas where no data within the viewing region were collected. (a) The number of reconnection events seen in each sector. (b) The number of hours Cassini spent inside the viewing region within that sector. (c) The number of reconnection events normalized to observation time.

As outlined in section 2.3 a definition of the “viewing region” is required when exploring the frequency of events observed. This study defines the viewing region as any location on the nightside of the planet, at a distance of more than 15*R*_*S*_ from Saturn, within the magnetosphere, outside of the area of Titan's influence, and not concurrent with a spacecraft SCAS maneuver. Discarding data not fulfilling these criteria leaves a total of 415 days worth of magnetometer observations. This equates to an average reconnection rate of 5.0 events or 1.4 clusters/episodes of reconnection per day. However, this does not take into account the different seasons and latitudes explored during this time. For example, during Southern Hemisphere summer when Cassini was closer to the hinged plasma sheet (in the later half of 2006), a subset of which is seen in Figure 5b, the reconnection rate reached 10.2 events per day.

#### 5.1.2 Event Frequency Across the Magnetotail

The occurrence of reconnection‐related events will be further examined. Figure 5a shows the spatial distribution of the number of events detected including plasmoids, TCRs, and dipolarizations. Figure 5b shows the coverage of Cassini inside the viewing region during the selected periods, and Figure 5c combines these to show the number of events normalized to the observing time within each sector. It is important to note that these figures are projected on the equatorial plane of the KSM system, and the latitude of the orbits and the magnetospheric environments encountered are not accounted for.

We note that the viewing region, as defined in section 2.3 above and illustrated in Figure 5b, includes broad magnetotail coverage and thus incorporates both lobe and plasma sheet intervals. Figure 5b shows that a large proportion of the data selected for this study lie on the dusk flank, namely, those orbits performed during 2009 and 2010. Meanwhile, Figure 5c is particularly useful when attempting to understand if a large number of event detections seen in Figure 5a reflect an increase in the occurrence frequency of events or is due to the large amount of time Cassini spent sampling that region.

Figure 5a displays a large number of events around dusk and midnight (during the symmetric midnight orbits near the plasma sheet), with considerably lower numbers seen on the dawn flank. The differences between the dawn and dusk regions are slightly reduced when the orbit occupation time is taken into consideration (in Figure 5c), where it appears that the majority of the events occur around or slightly post midnight. It is unclear from these figures whether the lower frequency of events on the dawn flank is due to inherent differences on the dawn flank or a result of the low latitude and lobe occupation of these orbits (see Figure 4a for an example of typical orbits during these intervals). As previously recognized, the orbits around the dawn flank (recorded during the early part of 2006) were made at a lower latitude and so were farther from the position of the hinged plasma sheet during the Southern Hemisphere's summer. This is reflected in the large proportion of the trajectory that is colored blue, indicating that Cassini was most likely within the lobe (from the criteria in section 2.4). The trajectory of the more symmetric midnight orbits, which observed the higher frequency of reconnection related events, is mostly colored green, indicating likely residence within or close to the plasma sheet. This would suggest that the difference in observation frequency could be, at least in part, due to the different viewing conditions found during the orbits.

**Figure 6:**
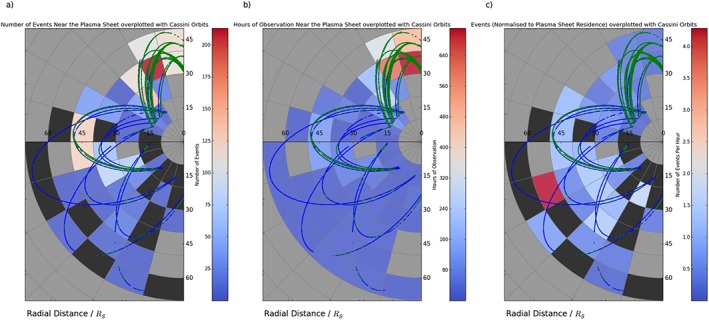
Color coded maps with 10*R*_*S*_ radial and 1 h local time bins. Maps are shown projected on the equatorial plane of the KSM system, with the Sun off to the right. Superimposed on the graph are the orbital trajectories of Cassini for 2006, late 2009, and 2010, within the viewing region. The blue sections of the trajectory represent times identified as within the lobe, while the green orbits outline times when Cassini is closer to or within the plasma sheet (from the criteria in section 2.4). Black regions show sectors where data were collected near or within the plasma sheet, but no events were detected. Meanwhile, grey segments show areas where no data near the plasma sheet were collected. (a) The number of reconnection events near the plasma sheet seen in each sector. (b) The number of hours Cassini spent near or within the plasma sheet in that sector. (c) The number of reconnection events (near the plasma sheet) normalized to observation time near the plasma sheet.

To explore this difference in more detail, Figure 6 looks solely at those events and parts of the orbit during which Cassini was located within or close to the plasma sheet (from the criteria in section 2.4). Figure 6a shows that as in Figure 5a, the majority of detections are made on the dusk flank or around midnight. Comparing this to Figure 6b, we can see that this correlates with the areas where Cassini spent the most time surveying the plasma sheet vicinity. Looking at the normalized rate in Figure 6c, we can see that when this is taken into account, the rate of detection peaks postmidnight. The drawback to this method is that plasma sheet occupation is not always independent of the event detections. In other words, the fact that the events themselves can cause the plasma sheet to bulge over spacecraft (changing the local conditions interpreted via the criteria in section 2.4) can have an effect on the observed occurrence rates. However, overall Figure 6c shows a steady increase in the frequency of event observations moving from dusk around to the postmidnight orbits. This could suggest that reconnection becomes more likely, as the stretched flux tubes rotate through midnight. A similar interpretation was made by *Thomsen et al.* [2013] in their survey of plasma data. Similarly, at Jupiter *Vogt et al.* [2014] found reconnection signatures most frequently in the dawn sector, though they note there could be other explanations for this.

We note that the trends discussed above persist if the bin sizes are adjusted.

#### 5.1.3 Event Occurrence: Tailward and Planetward Events

**Figure 7:**
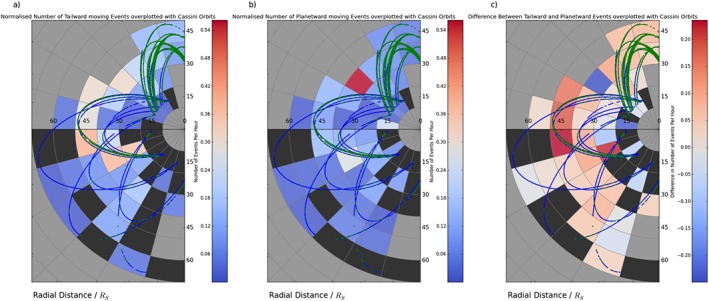
Maps comparing the frequency of (a) tailward and (b) planetward moving events with 10*R*_*S*_ radial and 1 h local time bins; event frequency is normalized to time spent in the viewing region within that sector. Maps are shown projected on the equatorial plane of the KSM system, with the Sun off to the right. Superimposed on the graph are the orbital trajectories of Cassini for 2006, late 2009, and 2010, within the viewing region. The blue sections of the trajectory represent time identified as within the lobe, while the green orbits outline times when Cassini is closer to or within the plasma sheet. Black regions show sectors where data was collected within the viewing region, but no events were detected. Meanwhile, grey segments show areas where no data within the viewing region was collected. Both Figures 7a and 7b are normalized to the same color scale for ease of comparison. (c) The map in Figure 7a minus the map in Figure 7b, the difference between the two.

Figures 7a and 7b show the same type of map as Figure 5c but for tailward and planetward moving detections, respectively. If the X line was typically located within a relatively narrow range of radial distance, then this might be expected to show planetward events preferentially occurring closer to the planet, with tailward events regularly found farther downtail (as has been observed at Jupiter [cf. *Vogt et al.*, 2010]). However, this is not strictly observed in the figure. Events inferred, from the sign of the *B*_*θ*_ deflection, to be planetward of the X line are observed at radial distances from 18 to 68.2*R*_*S*_, while those inferred to be tailward of the X line are observed at radial distances of 18 to 68.3*R*_*S*_. This spread of planetward and tailward detections suggests a variable X line. The inconsistent sampling conditions encountered by this study could also result in a such a spread and complicate the search for a statistical separatrix.

Figure 7c shows the difference between the maps in Figures 7a and 7b. Positive (red) values show sectors where more (normalized) tailward events are observed, while negative (blue) sectors show areas where planetward events are observed more often. The change from positive (red) to negative (blue) shows the region where the X line is most often located lies approximately between 20 and 30*R*_*S*_ (premidnight). If correct, this result would be approximately consistent with the modeling of *Jia et al.* [2012], which suggested a range of X line locations between 25 and 40*R*_*S*_ and the work of *Arridge et al.* [2015b] who reported an encounter with the diffusion region associated with reconnection at approximately 29*R*_*S*_. *Mitchell et al.* [2005] also suggested that reconnection could be playing a significant role in observed ion heating between 20 and 30*R*_*S*_. Though the results are broadly consistent, the presence of planetward moving events up to 68*R*_*S*_ downtail suggests that there could be other factors which control the X line location such as external solar wind changes or processes internal to the magnetosphere.

### 5.2 Event Morphology

The mean durations and *B*_*θ*_ deflections of the different classifications of events are shown in Table 3. TCR detections on both sides of the reconnection X line are observed to have a smaller Δ*B*_*θ*_ deflection than their plasmoid/dipolarization counterparts within the plasma sheet. This can be explained as TCRs are an indirect detection of the main event occurring within the plasma sheet.

Tailward moving TCRs are seen to have a larger average duration than plasmoids (those detections observed within the plasma sheet), the duration being the time between the northward and southward peaks. This could be explained as TCRs represent the draping of the magnetotail lobe field over the plasmoids (or dipolarizations), so they typify a wider disturbance rather than a direct encounter.

#### 5.2.1 Superposed Epoch Analyses of Events

**Figure 8:**
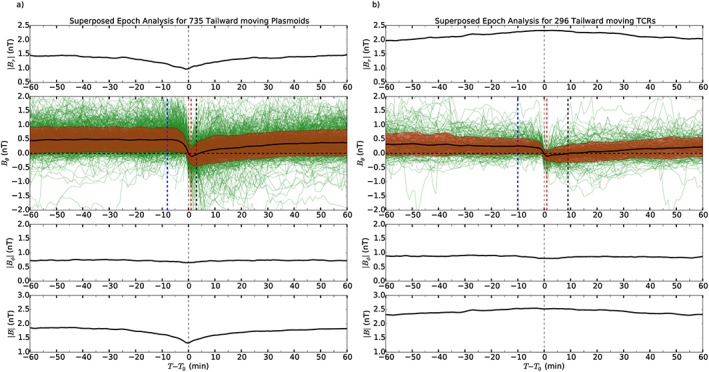
Superposed epoch analyses of (a) Plasmoids and (b) TCR events inferred to be tailward of the reconnection site. The magnetic field is given in the KRTP coordinate system, and the vertical scales are identical for both panels. The average traces for each component of the field are plotted with a thick black line. For the radial and azimuthal components the average absolute values are plotted as they can be positive or negative. The second panels (showing *B*_*θ*_) are double the size and range for both plots to illustrate the spread of field values. Also on the second panels, the faint green lines show the individual event traces that have been averaged, while the red shaded regions show the ±1*σ* extent of the results. The blue and red vertical dashed lines indicate the start and end of the event in the averaged trace, defined in the same way as in the algorithm, i.e., the largest maxima/minima either side of the event. The black dashed line marks the point at which the trace becomes southward again, the end of the (small) PPPS in Figure 8a.

Superposed Epoch Analyses (SEAs) of the event detections inferred to be tailward of the reconnection site can be seen in Figures 8a and 8b, which depict the results for 735 plasmoids and 296 TCRs, respectively. These do not represent the full catalog of events but only those which cross through *B*_*θ*_=0 (73% and 80% of the total detections, respectively). This has been done to facilitate comparison with previous studies, which had the requirement that the event pass through zero. The events have been aligned such that the point before which the trace becomes negative is at *t* = 0. The magnetic field is presented in KRTP coordinates, with the second panels (showing *B*_*θ*_) being double the height and range. The average traces are plotted with thick black lines, the first and third panels being the average of the absolute values of the *B*_*r*_, and *B*_*ϕ*_ components to ensure that they do not average to zero. In the *B*_*θ*_ panel the red shaded regions sketch the ±1*σ* extent of the results, while the individual event traces have been included in green to demonstrate that there is a significant spread in the signatures. The split between plasmoids and TCRs has been made using the lobe criteria detailed in equations (1) and (2) in section 2.4. As in the previous sections, any event during which the lobe criteria are violated is deemed to be a plasmoid in this classification scheme.

The first point of interest in both Figures 8a and 8b is that though the individual *B*_*θ*_ traces appear noisy before the event, the average (thick black line) corresponds well to the steady state small and southward field noted by *Jackman and Arridge* [2011] as characteristic of the background field in the magnetotail. Second, on average no sharp southward turn is seen at the start of events (in contrast to the idealized schematics in Figure 2) leaving only the northward turning. To explain this asymmetry, we suggest that events in our catalog are those linked to reconnection at a “near‐planet” X line. Scaling the near‐planet X line at Earth (typically found around ∼30*R*_*E*_) [*Imber et al.*, 2011] using the magnetopause standoff distances results in a predicted analogous near‐planet X line distance of ∼75*R*_*S*_ at Saturn. The terrestrial magnetosphere also contains a “distant” X line which typically lies ∼100+*R*_*E*_ downtail [*Slavin et al.*, 1985]; this scales to over 250*R*_*S*_ downtail at Saturn. If the picture in the Kronian magnetotail is similar to that at Earth, then the orbits performed by Cassini do not often reach far enough downtail to reach the distant X line [cf. *Eastwood and Kiehas*, 2015, Figure 16.1]. For a spacecraft trajectory which only samples the near‐planet region the asymmetry in the *B*_*θ*_ deflection results from the trajectory of the spacecraft through the magnetic structure, the effects of which have been discussed by *Borg et al.* [2012] and *Jackman et al.* [2014]. Furthermore, *Cowley et al.* [2015] argue that the asymmetric magnetic field signatures identified by *Jackman et al.* [2014] only represent the spacecraft's traversal of a small planetward section of the large extended plasmoid structure.

Figure 8a shows that the average SEA Δ*B*_*θ*_ for plasmoids is just under twice the size of the average SEA Δ*B*_*θ*_ for TCRs in Figure 8b (0.60nT compared to 0.37nT), as would be expected. This relationship is also seen in the mean values calculated from the catalog (discussed above and shown in Table 3).

In the average traces, as in the algorithm discussed in section 3.1, the start and the end of the event have been defined as the local north‐south maxima of the field. These have been indicated on Figures 8a and 8b by the blue and red vertical lines. For tailward moving plasmoids the start of the event is −8 min, and the end is at +1 min giving a duration of 9 min. The method of measuring duration is not particularly appropriate for this plot, as the lack of a significant southward peak means the start is not well defined. The same is true for Figure 8b, detailing the tailward moving TCRs, where the duration of the average event is found to be 11 min (from −10 to +1 min). In the same plot, the compression of the TCR in the |*B*| component of the field is observed to be much longer lived, up to 80 min. This could indicate that plasmoid release occurs, on average, ∼20–30 min prior to the TCR event time.

The top (*B*_*r*_) panels of Figures 8a and 8b both show consistent values of *B*_*r*_ before and after the event disturbance. This average background *B*_*r*_ can be seen to be around 1.4nT for plasmoids and 2.0nT for TCRs (representative of the lobe). At the center of the current sheet *B*_*r*_=0, in line with previous studies, we can use |*B*_*r*_| as a rough proxy for distance from the current sheet, assuming Harris sheet geometry [*Runov et al.*, 2006; *Arridge et al.*, 2008; *Jackman et al.*, 2011]. By this reasoning it can be confirmed that TCRs are, on average, seen at a greater distance from the center of the current sheet, as would be expected.

The average TCR signature, shown in Figure 8b, shows a change in the value of |*B*| during the event of ∼8%. This is approximately consistent with the 1–10% typical compression ratios reported at Earth [*Slavin et al.*, 1993, 2005]. The vertical extent of plasmoids, as inferred by their impact parameter, will be the focus of future work to examine the interior morphology of these structures.

The |*B*_*ϕ*_| panel of Figure 8a shows no major deviations during the average plasmoid passage. This may be consistent with a loop‐like picture of plasmoid field structure, as opposed to a flux rope‐type arrangement. Additionally, the |*B*| component of the field shows a marked reduction in the field during the event, again symptomatic of loop‐like structures (rather than flux rope type).

The average trace in Figure 8a does not display a long period where *B*_*θ*_ remains northward following the designated end of the event, in contrast with previous studies. The extended northward interval has been previously observed and interpreted as a post plasmoid plasma sheet (PPPS) [*Richardson et al.*, 1987; *Jackman et al.*, 2011, 2014]. This is an interval during which open flux in the magnetotail is closed following plasmoid passage down the magnetotail. Previously, *Jackman et al.*[2011] observed a 58 min long northward interval following the average event, while *Jackman et al.*[2014] reported 27 min more recently based on a larger catalog from the deepest tail orbits of 2006. In contrast, Figure 8a only shows a 2 min northward excursion. If the sample of events plotted is limited to those found in 2006 (the same time period as explored by *Jackman et al.*[2014]), then this increases to a 5 min interval. The simplest explanation for the absence of a large statistical PPPS in this new catalog is the inclusion of many smaller events (in terms of Δ*B*_*θ*_ and duration); the largest deflections of the field are easier to spot and therefore more likely to have been selected in the past. Furthermore, previous by eye studies were perhaps more likely to pick out events with a significant PPPS, as they would stand out more.

Though there is no PPPS as such in Figure 8a, the gentle sloping recovery (whereby the field takes 40 to 50 min to return to background levels) is consistent in shape to the asymmetry reported by *Jackman et al.* [2014].

## 6 Discussion

The combination of a larger data set encompassing a greater proportion of the Kronian magnetotail, including coverage during different seasons, and a new automated method ideal for identifying small scale reconnection related events allows insight into the global dynamics of the Kronian magnetosphere.

### 6.1 Mass Budget

We now examine the contribution of the inferred reconnection events to mass circulation at Saturn. There are three main ways in which plasma can enter the magnetosphere: from the solar wind, from the Kronian ionosphere, and from ionized neutrals originating from the moons and rings (See *Blanc et al.* [2015] for a review). The main plasma source for the Kronian magnetosphere is the moon Enceladus, from which the majority of the water group ions originate [e.g., *Hansen et al.*, 2006; *Sittler et al.*, 2008]. The rings also contribute a small fraction of the detected water group ions, while Titan is a (smaller) source of nitrogen and hydrogen ions [*Smith et al.*, 2007, 2008; *De La Haye et al.*, 2007], including a significant amount of H_2_+ ions [e.g., *Thomsen et al.*, 2010]. Light ions are generally thought to originate from either Saturn's ionosphere, Enceladus, Titan, or the solar wind, depending on their location within the magnetosphere [*Glocer et al.*, 2007; *Felici et al.*, 2016]. The majority of the neutrals produced from the rings or moons escape the Kronian system, but between ∼10% [*Fleshman et al.*, 2010] and 30% [*Jurac and Richardson*, 2005] are ionized, leading to a plasma mass loading rate of between 10 and 220kgs^−1^. In order to lose just 100kgs^−1^, *Bagenal and Delamere* [2011] calculated that plasmoids would need to be ejected at a rate of 200 per day. This calculation was based on a plasmoid volume of (10*R*_*S*_)^3^ with a density of 0.01cm^−3^ (18 amu ions).

As we lack reliable plasma moments for all events in our new catalog, we take a conservative value of 300kms^−1^ for the plasmoid velocity (based on the mean value found for 29 events in the *Jackman et al.* [2014] study). We incorporate this velocity estimate and combine it with the range of durations from our new catalog and find a range of plasmoid lengths between 0.30 and 10.45*R*_*S*_. For the height of the plasmoid we use a value of 7*R*_*S*_ (as in *Cowley et al.* [2015]) to account for the bulging of the plasma sheet (whose quiescent thickness has been observed to be around 4*R*_*S*_ [*Kellett et al.*, 2009; *Arridge et al.*, 2011; *Sergis et al.*, 2011; *Szego et al.*, 2012]). A value of 80*R*_*S*_ has been assumed as the upper limit to the azimuthal extent of a plasmoid. This represents the approximate width of the Kronian tail at *X*_KSM_=−40*R*_*S*_ from the model of *Pilkington et al.* [2015] (with a relatively high solar wind dynamic pressure of 0.1nPa). Combining these values with a density of 0.1cm^−3^ (16 amu ions) [*Thomsen et al.*, 2014] gives a range of plasmoid masses between 9.8 × 10^4^kg and 342 × 10^4^kg. These masses correspond to a required plasmoid loss rate of between 2.5 and 88 per day, in order to explain the loss of 100kgs^−1^.

Though an average occurrence rate of 10.2 events per day was observed during the midnight orbits, which is within the lower estimate required to balance the Enceladus input, it is highly unlikely that the bulk of events found fulfill the large azimuthal extent incorporated into the above calculation. MHD modeling by *Zieger et al.* [2010] and *Jia et al.* [2012] suggest that large‐scale plasmoids may only be responsible for around 8%–10% of the total mass loss, around 0.8kgs^1^ to 22kgs^1^, a total that could be explained or exceeded by the observations in this work. In this scenario the majority of the mass is lost through smaller‐scale mechanisms, such as cross‐field diffusion [*Bagenal and Delamere*, 2011] or along the flanks [*Kivelson and Southwood*, 2005; *Jia et al.*, 2012].

At Earth various studies have estimated the azimuthal width of flux ropes (plasmoids) resulting from tail reconnection, and results range from 15*R*_*E*_ [*Slavin et al.*, 1993] to 40*R*_*E*_ [*Ieda et al.*, 1998]. In general, observations agree that they do not fill the entire width (∼48*R*_*E*_) [*Fairfield*, 1992] of the magnetotail [*Kiehas et al.*, 2013]. Additionally, the lack of a one‐to‐one correlation between substorms and flux rope observations supports the notion that flux ropes are limited in their azimuthal range [*Nagai et al.*, 1994]. Therefore, the use of the approximate full tail width, 80*R*_*S*_, as the upper limit to the azimuthal extent will lead to an overestimation of the mass contained within the plasmoid structures. However, it is not possible to determine the true azimuthal extent with a single spacecraft, so the numbers calculated with this must be viewed as an upper limit in this regard. The azimuthal extent of the structures also has ramifications as to the observed frequency of events; if plasmoids are tightly azimuthally confined, then many will be missed by a single spacecraft. As noted by *Cowley et al.* [2015] these factors cancel, given isotropic observation and occurrence.

Another important caveat to consider in this interpretation is the uncertainty in the plasmoid length. The definition of duration used by the algorithm (the time between northward and southward extrema) [*Slavin et al.*, 1993] has been shown in the past to underestimate plasmoid size by a factor of ∼4 to 8 [*Kivelson and Khurana*, 1995]. Additionally, the plasma signature of events observed by *Jackman et al.* [2014] had a longer duration than the magnetic field signature. Indeed, recent work, including *Arridge et al.* [2015b], *Jackman et al.* [2015], and *Thomsen et al.* [2015], has suggested that reconnection related flows can last for many hours.

The MHD simulations of *Jia et al.* [2012] produced plasmoids with an approximately circular cross section of radius ∼10*R*_*S*_, giving a length of ∼20*R*_*S*_, also somewhat larger than our estimate. Recently, *Cowley et al.* [2015] suggested from geometrical arguments that the observed asymmetric magnetic field signatures identified by this study represent only a small proportion of the full plasmoid structure. Therefore, the method used above to calculate the plasmoid length significantly underestimates their full length. During the 4.8 h between events, corresponding to the average occurrence rate of 5.0 per day observed by this study, and using the arguments of *Cowley et al.* [2015], it is possible to calculate that the full stretched plasmoid length could be around 30*R*_*S*_. This estimation assumes that the events in the catalog are isolated; however, as discussed in section 4 some of these events could be related to the same reconnection episode (e.g., secondary island detections in the interval discussed by *Arridge et al.* [2015b]). For this reason, the true stretched length could be larger than this and in any case significantly greater than the 0.3 to 10.45*R*_*S*_ calculated from the magnetic field deflections. These length arguments could help reconcile the imbalance between the observed and required mass loss.

### 6.2 Flux Closure and Reconnection Cycles

Previous studies linking the change in size of the auroral oval to the varying magnetic flux contained in the polar cap [e.g., *Badman et al.*, 2005, 2014] have highlighted the importance of reconnection both at the magnetopause and in the magnetotail. It is possible to estimate the flux closed in a reconnection event using the duration of the northward interval, interpreted as a post plasmoid plasma sheet (PPPS). Unlike previous studies, Figure 8a does not show a statistical average extended northward period (discussed in section 5.2.1), which we have suggested is a result of the different selection criteria and the inclusion of large numbers of new events. If many of these new events do not show a distinct PPPS, they could represent the reconnection of purely closed flux, i.e., be related to the Vasyliunas cycle, and some may be detections of secondary islands which would not be expected to produce a PPPS.

Though no statistical PPPS is observed, some individual events do show considerable extended northward intervals. Twenty‐five events show a PPPS longer than 30 min, while six of these are longer than 1 h. This could suggest that the closing of flux in tail reconnection is sporadic and occurs primarily in fewer, large‐scale events.

We now compare the reconnection signatures observed in the dawn and dusk regions of the magnetosphere. Previous theoretical studies have suggested distinct locations in the magnetotail where the Dungey and Vasyliunas cycles preferentially operate [*Cowley et al.*, 2004; *Badman and Cowley*, 2007]. For this purpose SEAs have been produced using subsets of the observed plasmoid events. The subsets have been selected based on the local time at which the events were observed. The primary features of comparison in these plots are the lengths of the PPPS seen and depth of northward deflections.

#### 6.2.1 Comparing Dawn and Dusk

**Figure 9:**
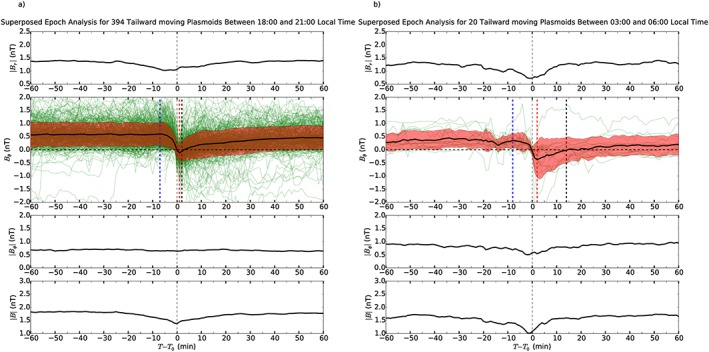
Superposed epoch analyses of events observed (a) postdusk and (b) predawn inferred to be tailward of the reconnection site. The format is the same as for Figure 8.

The SEAs shown in Figure 9 only include events that were observed within 3 h of dusk and dawn, respectively. The SEAs are plotted in the same format as Figure 8 above. When analyzing these plots, it is important to consider the differences in magnetospheric environment encountered by Cassini during these orbits. For example, the 3 h window predawn (03:00 to 06:00) contains orbits performed during 2006. Figure 1 shows that these orbits were all performed at approximately the same equatorial latitude (between 0° and 0.5°); during Southern Hemisphere summer this latitude was below the position of the hinged plasma sheet for the majority of the time, similar to position 3 in Figure 2a. In comparison the orbits in the dusk sector (18:00 to 21:00) were mainly obtained during 2009 and 2010 (around Kronian equinox), in locations where Cassini spent over 50% of its time close to or within the plasma sheet (from Table 1).

Figure 9b reveals the presence of a significant (12 min) PPPS, while Figure 9a shows no such long northward interval. From this figure it could be concluded that the events around dawn are more likely to involve the closure of open field.

However, there are a few background considerations to take into account. First, only 20 plasmoid‐like events were observed within the selected dawn local time sector, compared to 394 events in the 3 h around dusk. The effect of this can be seen in the less steady average traces observed in all four panels of the dawn SEA. Second, as previously mentioned, the orbits around dawn were low latitude, equatorial orbits performed during Southern Hemisphere summer; Cassini spent 92% of this period within the lobe (Table 1). Therefore, it is possible that the 20 events sampled by Cassini during this time correspond to larger events, perhaps those most likely to involve the closure of open field.

The Kolmogorov‐Smirnov test can be used to compare two samples, with the null hypothesis that the samples are drawn from the same distribution. Applying the test to the distributions of PPPS duration observed results in a *p* value of 0.209, with a K‐S statistic of 0.236. These numbers suggest that the null hypothesis cannot be rejected and that the two samples could be from the same distribution. Therefore, the difference in PPPS lengths could be a sampling effect; a much larger sample at dawn would be required to show otherwise.

It is also worth noting that when the local time bins are expanded to cover the full width of the magnetotail (with the two local time bins being separated at midnight), the distributions of PPPS length are very similar (with a K‐S statistic and *p* value of 0.74 and 0.055, respectively). Additionally, when the local time distribution of the 25 events with considerably large PPPS (greater than 30 min) is analyzed, it is found that the proportion of event detections that these represent premidnight and postmidnight is approximately equal (∼3–4%).

## 7 Conclusions

We have presented a new catalog of reconnection‐related events, identified using an algorithm which automatically selects deflections in the meridional (*B*_*θ*_) component of the magnetic field. In order to be selected, the events must fit a polynomial to a sufficient quality, and the size of the deflection must be greater than 1.5× the local RMS of *B*_*θ*_ (and be greater than 0.25nT). The orientation of the change was related to the position of the spacecraft relative to the X line. The events were also confirmed by eye. The new catalog covers data taken by the Cassini spacecraft over three years: 2006, 2009, and 2010. This combination allows us, for the first time, to explore the asymmetry between dawn (2006) and dusk (2009 and 2010) in terms of the magnetic field signatures associated with reconnection.

When normalized to observation time, reconnection is observed most frequently around and postmidnight, with more infrequent but steady loss seen on the dusk flank. The increasing occurrence frequency as the mass loaded flux tubes rotate from dusk past midnight perhaps indicates the increase in instability as they stretch down the magnetotail. Observations on the dawn flank took place largely while the spacecraft was in the southern magnetotail lobe, which often precluded the direct observation of plasmoids and dipolarizations but allowed for observations of TCRs. This data set shows sporadic and clustered reconnection. The observed frequency of reconnection events is seen to change dramatically depending on the latitude of the observations (relative to the position of the hinged plasma sheet). For example, the frequency is relatively low during intervals when Cassini explored the magnetotail lobes. Normalizing the occurrence to plasma sheet occupation confirms an increased frequency of observation postmidnight. The reconnection X line position appears to be highly variable, with events inferred to be both planetward and tailward of the X line observed at most radial distances and local times. Overall, however, more planetward moving events are seen at distances closer than 30*R*_*S*_, suggesting that the reconnection X line is often located in this vicinity.

The average post plasmoid plasma sheet (PPPS) observed following plasmoids in previous studies is not observed with this catalog. This could be a result of the different selection criteria and inclusion of many more events with smaller deflections (shown by the smaller average deflection size). Due to a limited sample of events near dawn, it is not possible to determine conclusively whether flux is closed preferentially premidnight or postmidnight, though it is likely that other factors, such as the solar wind conditions, play a role. The upper limit to the mass loss calculated from the catalog alone is insufficient to balance the input from Enceladus. Potential solutions to this disparity include mass loss from other processes [*Kivelson and Southwood*, 2005; *Zieger et al.*, 2010; *Bagenal and Delamere*, 2011; *Jia et al.*, 2012; *Sergis et al.*, 2013] or that the durations used in this study underestimate the plasmoid size and therefore mass (due to the trajectory of Cassini through the structures) [*Kivelson and Khurana*, 1995; *Cowley et al.*, 2015].
